# Transmission of Single and Multiple Viral Variants in Primary HIV-1 Subtype C Infection

**DOI:** 10.1371/journal.pone.0016714

**Published:** 2011-02-09

**Authors:** Vladimir Novitsky, Rui Wang, Lauren Margolin, Jeannie Baca, Raabya Rossenkhan, Sikhulile Moyo, Erik van Widenfelt, M. Essex

**Affiliations:** 1 Department of Immunology and Infectious Diseases, Harvard School of Public Health AIDS Initiative, Harvard School of Public Health, Boston, Massachusetts, United States of America; 2 Botswana–Harvard AIDS Institute, Gaborone, Botswana; 3 Department of Biostatistics, Harvard School of Public Health, Boston, Massachusetts, United States of America; Institut Pasteur, France

## Abstract

To address whether sequences of viral *gag* and *env* quasispecies collected during the early post-acute period can be utilized to determine multiplicity of transmitted HIV's, recently developed approaches for analysis of viral evolution in acute HIV-1 infection [1], [2] were applied. Specifically, phylogenetic reconstruction, inter- and intra-patient distribution of maximum and mean genetic distances, analysis of Poisson fitness, shape of highlighter plots, recombination analysis, and estimation of time to the most recent common ancestor (tMRCA) were utilized for resolving multiplicity of HIV-1 transmission in a set of viral quasispecies collected within 50 days post-seroconversion (p/s) in 25 HIV-infected individuals with estimated time of seroconversion. The decision on multiplicity of HIV infection was made based on the model's fit with, or failure to explain, the observed extent of viral sequence heterogeneity. The initial analysis was based on phylogeny, inter-patient distribution of maximum and mean distances, and Poisson fitness, and was able to resolve multiplicity of HIV transmission in 20 of 25 (80%) cases. Additional analysis involved distribution of individual viral distances, highlighter plots, recombination analysis, and estimation of tMRCA, and resolved 4 of the 5 remaining cases. Overall, transmission of a single viral variant was identified in 16 of 25 (64%) cases, and transmission of multiple variants was evident in 8 of 25 (32%) cases. In one case multiplicity of HIV-1 transmission could not be determined. In primary HIV-1 subtype C infection, samples collected within 50 days p/s and analyzed by a single-genome amplification/sequencing technique can provide reliable identification of transmission multiplicity in 24 of 25 (96%) cases. Observed transmission frequency of a single viral variant and multiple viral variants were within the ranges of 64% to 68%, and 32% to 36%, respectively.

## Introduction

Complexity and multiplicity of HIV-1 transmission depends on multiple factors, although HIV-1 subtype [3] and mode [2], [4], [5] of viral transmission can be considered major determinants. A severe genetic bottleneck during heterosexual transmission of HIV-1 subtype C has been reported [3], [6]. Compelling evidence for a link between multiplicity and mode of HIV-1 transmission was provided by a series of recent studies that applied the technique of single-genome amplification/sequencing (SGA) of samples collected at the very early clinical stage of HIV-1 infection, and combined this method with a model of random viral evolution as a new tool for assessment of HIV-1 transmission multiplicity [1], [2], [4]–[7]. Transmission of a single viral variant occurs in about 76–90% of cases of heterosexual transmission [2], [6], [7], in about 60% of cases of HIV-1-infected men who have sex with men (MSM) [5], and only in about 40% of injection drug users (IDU) who acquired HIV-1 infection [4]. Conversely, transmission of multiple viral variants gradually increases from about 20% during heterosexual transmission of HIV-1 to about 40% in MSM, and to 60% in IDU. It is likely that the mucosal barrier plays an important role in reducing multiplicity of transmitted HIV-1. Due to the absence of a mucosal barrier, IDU exhibit a higher frequency of multiple-variant transmission and a wider range of transmitted viruses than subjects infected heterosexually [4]. The important role of the mucosal barrier in viral transmission has also been demonstrated in rhesus macaque models [8], [9].

Transmission of multiple viral variants is associated with faster disease progression [10]–[12]. Haaland et al. reported transmission of multiple viral variants in 3 of 7 individuals infected by someone other than their spouses, and significant association between transmission of multiple variants and an inflammatory genital infection [6]. Therefore, monitoring the multiplicity of new HIV-1 transmissions is important for assessing the efficiency of public health interventions including design and development of therapeutic and preventive strategies, and interventions targeting behavior change. However, the current tools for identifying multiplicity of transmitted viruses in new HIV-1 infections are suboptimal for routine monitoring.

The limited number of analyzed cases has been an inherent limitation in most primary HIV-1 infection studies due to the numerous logistical challenges in obtaining clinical samples during the acute and early post-acute phases of HIV-1 infection. Thus, studies addressing multiplicity of HIV-1 infection included 28 MSM from New York, Alabama, and North Carolina [5], 102 HIV-infected blood donors and healthcare patients from the USA and Trinidad [2], 69 individuals from South Africa and Malawi [7], 27 heterosexually infected individuals from Zambia and Rwanda [6], and 10 IDU in a Montreal cohort [4].

In this study we addressed whether viral sequences obtained within 50 days post-seroconversion (p/s) can be utilized for assessing the multiplicity of viral transmission in primary HIV-1 subtype C infection. The *gag* and *env* gp120 quasispecies generated by SGA from a cohort of acutely and recently HIV-1C-infected individuals in Botswana were studied. Applying recently developed techniques for analysis of viral evolution in acute HIV-1 infection [1], [2], a two-step approach was explored to assess multiplicity of HIV transmission. Congruent results were obtained for 96% of analyzed samples.

## Methods

### Ethics statement

This study was conducted according to the principles expressed in the Declaration of Helsinki. The study was approved by the Institutional Review Boards of Botswana and the Harvard School of Public Health. All patients provided written informed consent for the collection of samples and subsequent analysis.

### Study subjects

Viral sequences analyzed in the study originated from the Primary HIV-1 Subtype C Infection Study in Botswana, the *Tshedimoso* study [13]–[18]. A total of 25 individuals with estimated time of seroconversion and samples collected within 50 days p/s were used in this study. In the description of study subjects the following terminology was followed: *acute* HIV infection was defined as the period from viral transmission to seroconversion; *recent* HIV infection was defined as the period from seroconversion until one year p/s; *primary* HIV infection included both the periods of *acute* and *recent* HIV infection. The time interval of 0–50 days p/s was a primary focus in this study, and was termed the *early post-acute period*. Subjects' characteristics at the time of sampling, including gender, age, HIV-1 RNA load, proviral DNA load, CD4 count, timing of sample collection, and the number of analyzed *gag* and *env* sequences, are presented in Table 1. The time of seroconversion was estimated based on the laboratory results of HIV-1 RNA test, ELISA test for HIV antibodies, and Western blot test as described previously [14], [15]. Eight *acutely infected* individuals (subjects A to H) were identified before seroconversion and the time of their seroconversion was estimated as the midpoint between the last ELISA-negative and the first ELISA-positive test, within a week in most cases. Thirty-four *recently infected* individuals were identified after seroconversion, and the time of their seroconversion was estimated according to Fiebig staging [19] based on incomplete Western blot. For example, subject OG's regular ELISA test for HIV antibodies was positive indicating seroconversion, while her detuned ELISA test was negative suggesting recent HIV infection. The Western blot analysis revealed presence of the gp160-, gp120-, gp41-, p66-, p55/51-, and p24-bands, and absence of p39-, p31-, and p17-bands. Plasma HIV-1 RNA was 6.47 log_10_/ml, and gradually declined over the next few months. Therefore, the analyzed sample of subject OG was classified as Fiebig stage V. The beginning of Fiebig stage III coincides with the time of detectable seroconversion (time 0). Given that the mean duration of Fiebig stage III is 3 days, the mean duration of Fiebig stage IV is 6 days, and the mid-point of mean duration of Fiebig stage V is 35 days, the enrollment time for subject OG was assigned 44 days p/s. Details on quantification of viral load and CD4 [13], [17], [18], and amplification and sequencing of viral quasispecies by SGA [13], [15], [16] have been presented elsewhere. Analyzed sequences were tested by HYPERMUT v.2.0 [20] and hypermutated sequences that yielded a p-value of 0.05 or lower were excluded from analysis. All subjects were Botswana nationals, and all infections were HIV-1 subtype C [13], [17]. All subjects were ARV-naïve at the time of sampling.

**Table 1:** Patient characteristics, time of sampling, and number of analyzed *gag* and *env* sequences

						Time of sampling			Sequences, n	
Patient code	Gender	Age^1^	HIV-1 RNA load^2^	Proviral DNA load^3^	CD4 count	Date^4^	Days from estimated s/c^5^	Fiebig stage^6^	*gag*	*env*
A-1811	F	31	6.09	2.37	172	04/13/2004	6 and22^7^	II	16	19
B-2865	F	20	5.74	3.50	373	08/30/2005	10 and18^7^	II	11	33
C-3312	F	32	6.56	3.09	202	12/07/2005	4	II	36	30
D-5018	M	27	6.32	2.73	286	11/14/2006	6	II	29	22
E-3430	F	35	1.70	2.21	640	02/07/2006	30	II	24	12
F-3505	F	53	5.57	3.19	426	04/11/2006	7	II	7	13
G-3603	M	34	5.76	1.78	505	06/30/2006	4	II	10	11
H-5582	F	26	3.36	3.88	442	06/10/2007	16	II	9	12
OC-2381	M	25	5.08	3.27	301	02/15/2005	27	IV	16	7
OG-2604	F	23	6.47	3.37	260	06/27/2005	44	V	12	15
OJ-2761	F	26	3.89	3.62	438	08/04/2005	44	IV	16	11
OU-3091	M	27	4.95	1.24	302	10/17/2005	13	IV	10	11
OW-3234	M	41	5.41	2.81	234	11/16/2005	13	IV	8	11
O1-3354	F	30	1.99	2.27	578	12/16/2005	6	IV	10	12
PK-4872	M	30	3.27	1.29	698	08/31/2006	47	V	7	8
PO-5062	F	20	2.88	1.25	460	12/13/2006	6	IV	10	6
PP-5065	F	23	6.29	2.68	319	12/15/2006	44	V	10	8
QA-5099	M	28	5.01	3.61	305	01/11/2007	6	IV	11	13
QI-5715	F	27	5.59	2.57	292	07/18/2007	20	IV	10	16
QJ-5768	F	29	3.03	2.37	509	08/02/2007	8	IV	4	-
QM-5849	F	37	1.70	1.81	879	08/28/2007	48	V	6	7
QP-5867	M	29	2.74	2.71	626	09/03/2007	48	V	10	14
QR-5943	F	26	2.60	1.75	801	09/25/2007	7	IV	9	16
QS-6020	F	26	2.60	1.73	1,357	10/19/2007	44	V	8	7
QT-6024	F	24	4.89	2.53	522	10/22/2007	44	V	11	21

1 Years at the time of sampling.

2 log_10_ copies/ml.

3 log_10_ copies/10^6^ PBMC.

4 Date of sampling (first sampling for patients with dual dates of sampling).

5 Seroconversion.

6 Fiebig EW, Wright DJ, Rawal BD, Garrett PE, Schumacher RT, et al. (2003) Dynamics of HIV viremia and antibody seroconversion in plasma donors: implications for diagnosis and staging of primary HIV infection. AIDS 17: 1871-1879.

7 Subjects A and B had viral sequences available at two time points within 50 days p/s. Both sets were included in analysis.

### Phylogenetic analysis

The branching topology of intra-patient *gag* and *env* sequences was inferred by the Neighbor-Joining (NJ) method (Tamura-Nei model with bootstrapping) and the Maximum-Likelihood (ML) method (PhyML) as implemented by Geneious v.5.0.3 [21]. Phylogenetic trees were visualized in FigTree [22].

### Analysis of viral distances

Viral pairwise ML-corrected distances were analyzed using DIVEIN [23]. The Kimura-2-parameters (K2P)-corrected and Hamming distances were analyzed in MEGA v4 [24]. The MRCA and pairwise ML-corrected distances to MRCA were estimated in DIVEIN [23]. The MRCA, a hypothetical viral sequence that represents the most recent viral variant from which a subject's viral quasispecies are descended, was reconstructed in DIVEIN [23] by the joint maximum likelihood procedure [25]. The majority consensus sequence, another hypothetical viral sequence that indicates the most abundant nucleotide in the multiple sequence alignment at each position, was built in BioEdit [26]. The pairwise K2P-corrected and Hamming distances to the consensus sequence were quantified in MEGA v4 [24].

### Poisson fitness

Poisson fitness analysis was performed using the online tool at Los Alamos National Laboratory at http://www.hiv.lanl.gov/content/sequence/POISSON_FITTER/poisson_fitter.html [27]. The tool analyzes frequency of Hamming distances by computing the best fitting Poisson distribution through ML and evaluating results of the Goodness of Fit test (GOF).

### Highlighter plots

Highlighter plots were generated by Highlighter at www.hiv.lanl.gov, a visualization tool of aligned nucleotide sequences that highlights nucleotide polymorphisms and marks APOBEC signatures.

### Recombination analysis

Recombination analysis was performed using package RDP3, a computer program for statistical identification and characterization of recombination events in DNA sequences [28]. RDP3 utilizes a range of non-parametric recombination detection methods including BOOTSCAN, MAXCHI, CHIMAERA, 3SEQ, GENECONV, SISCAN, PHYLPRO and VISRD [29]–[34]. RDP3 treats every sequence within the analyzed alignment as a potential recombinant, and systematically screens sequence triplets and/or quartets to identify sequences that contain a recombinant and two sequences that could serve as parents, and performs a statistical evaluation of recombination signal [28]. Such an approach eliminates the need for reference sequences, which makes analysis of viral quasispecies from epidemiologically unlinked patients more practical.

### Estimation of tMRCA

Analysis was performed using Bayesian inference with a Markov Chain Monte Carlo (MCMC) method implemented in BEAST v.1.5.4 [35]. Longitudinal viral quasispecies dated according to the day of sampling from a subset of six acutely infected individuals were utilized to identify the rate of viral evolution within HIV-1 subtype C *gag* and *env*. For acutely infected individuals, the MRCA of viral quasispecies sampled at a given time was constrained to a uniform calibration prior bounded between the time of sample collection in relation to the estimated time of seroconversion (lower bound) and the same value plus 30 days as an average time period between the time of infection and seroconversion. The rate of evolution over the entire tree was estimated as the meanRate parameter for each case of acute HIV-1 infection. The geometric mean evolutionary rate in HIV-1C *gag* was estimated at 1.23E-05 (95% CI 8.07E-06 – 1.64E-05) substitutions per site per day. The geometric mean evolutionary rate in HIV-1C *env* gp120 was estimated at 3.71E-05 (95% CI 1.97E-05 – 5.45E-05) substitutions per site per day. The estimated rate of *gag* and *env* gp120 evolution was applied to determine tMRCA for five “undetermined” cases in the study, and the tMRCA was estimated as the treeModel.rootHeight parameter. The *gag* and *env* gp120 sequence data were analyzed using the evolutionary model selected by the Akaike information criterion in jModeltest 0.1.1 [36] and a relaxed molecular clock (uncorrelated lognormal) under the Yule model. The value of the effective sampling size (ESS) was controlled to be above 200, and the length of the MCMC chain was at least 20,000,000.

### The decision on multiplicity of HIV infection

The decision on multiplicity of HIV infection was made based on the model's fit with or failure to explain the observed extent of viral sequence heterogeneity. The model's fit was associated with transmission of a single viral variant, while the model's failure was interpreted as either transmission of multiple viral variants, or the result of rapid immune selection driving the observed level of viral diversification [1], [37]–[39].

## Results

### Heterogeneity of HIV-1 subtype C *gag* and *env* sequences

**Figure 1:**
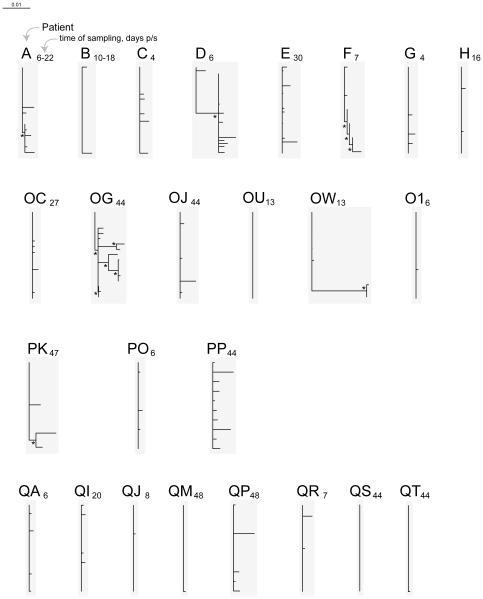
HIV-1 subtype C diversity within 50 days p/s among acutely and recently infected individuals from the Tshedimoso cohort in Botswana: *gag* sequences, NJ tree. Acutely infected individuals are denoted by a single letter A through H. The patient ID of recently infected individuals has two letters, OC to QT. A subscript next to the patient ID denotes time of sampling in days p/s. Asterisks denote bootstrap values ≥80. The horizontal bar represents genetic distance.

**Figure 2:**
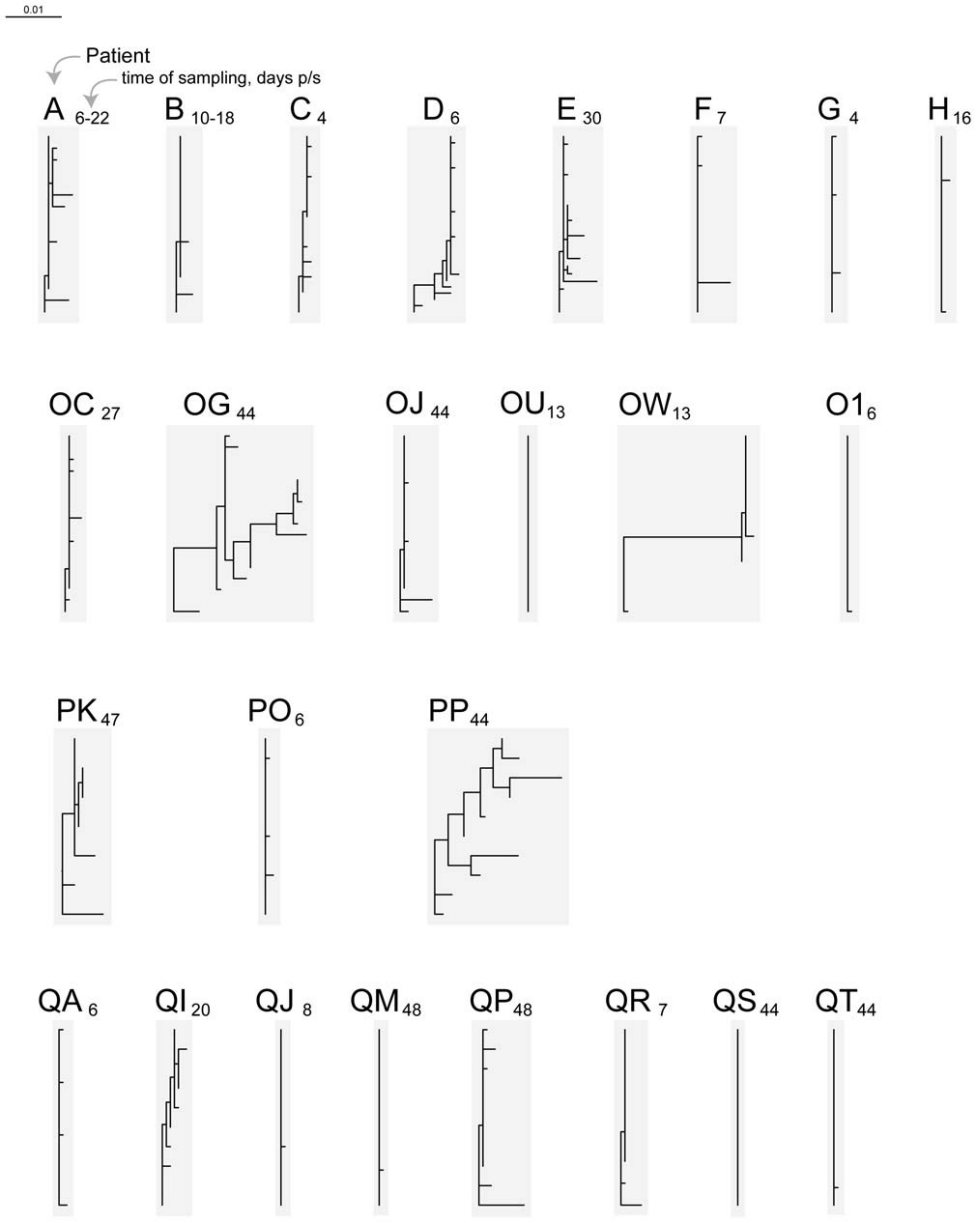
HIV-1 subtype C diversity within 50 days p/s among acutely and recently infected individuals from the Tshedimoso cohort in Botswana: *gag* sequences, ML tree. Acutely infected individuals are denoted by a single letter A through H. The patient ID of recently infected individuals has two letters, OC to QT. A subscript next to the patient ID denotes time of sampling in days p/s. The horizontal bar represents genetic distance.

**Figure 3:**
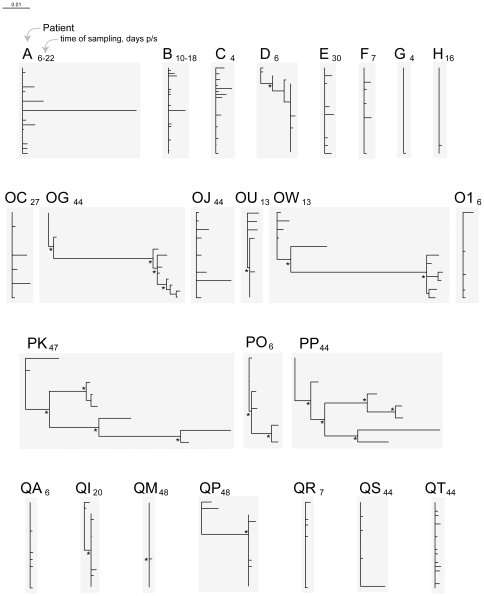
HIV-1 subtype C diversity within 50 days p/s among acutely and recently infected individuals from the Tshedimoso cohort in Botswana: *env* sequences, NJ tree. Acutely infected individuals are denoted by a single letter A through H. The patient ID of recently infected individuals has two letters, OC to QT. A subscript next to the patient ID denotes time of sampling in days p/s. Asterisks denote bootstrap values ≥80. The horizontal bar represents genetic distance.

**Figure 4:**
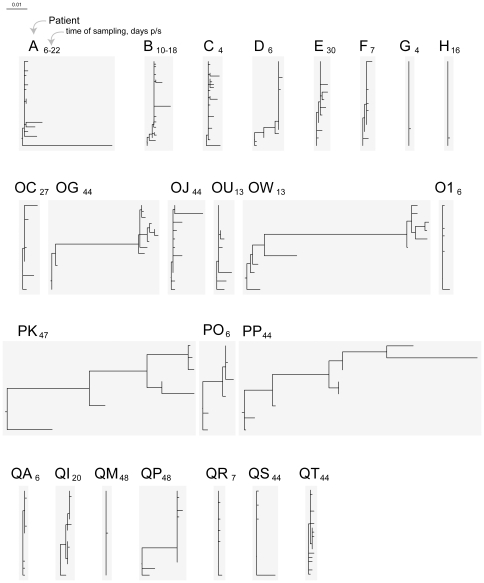
HIV-1 subtype C diversity within 50 days p/s among acutely and recently infected individuals from the Tshedimoso cohort in Botswana: *env* sequences, ML tree. Acutely infected individuals are denoted by a single letter A through H. The patient ID of recently infected individuals has two letters, OC to QT. A subscript next to the patient ID denotes time of sampling in days p/s. The horizontal bar represents genetic distance.

Using samples collected within 50 days p/s, *gag* quasispecies were generated for 25 and *env* for 24 HIV-infected individuals with estimated time of seroconversion (Fiebig stage less than VI). Patient demographic and laboratory data, time of sampling, and number of analyzed *gag* and *env* sequences at the time of sampling are presented in Table 1. The phylogeny of *gag* and *env* sequences was inferred by the NJ and ML methods. The overall length of branches was shorter for *gag* sequences (Fig. 1 – *gag* NJ tree, and Fig. 2 – *gag* ML tree) as presented on the same scale with *env* sequences (Fig. 3 – *env* NJ tree, and Fig. 4 – *env* ML tree), which is consistent with lower diversity of HIV-1 *gag* as compared with HIV-1 *env*.

### Branching topology of *gag* sequences

The differences among *gag* sequences were less discernible as compared to *env* sequences. Three subjects in the NJ tree (Fig. 1; subjects D, OG, and OW) and three subjects in the ML tree (Fig. 2; subjects OG, OW, and PP) demonstrated extended length of branches suggesting transmission of multiple viral variants, and highlighting the necessity of using alternative methods for inferring phylogenetic trees. Subjects A, D (ML tree), E, F, OJ, PK, PP (NJ tree), and QP (NJ and ML trees) demonstrated moderate diversity of *gag* sequences. Fifteen of 25 subjects demonstrated short (13 subjects) or zero (subjects OU and QS) branch length of *gag* quasispecies collected within 50 days p/s. Thus, branching length and topology of *gag* sequences was consistent with transmission of multiple viral variants in five (20%) cases (subjects D, OG, OW, PK, and PP), undetermined multiplicity of transmission in five (20%) cases (subjects A, E, F, OJ, and QP), and transmission of a single viral variant in 15 (60%) cases.

### Branching topology of *env* sequences

Branching topology and length of *env* sequences in both NJ (Fig. 3) and ML trees (Fig. 4) suggested transmission of multiple viral variants in 5 cases (subjects A, OG, OW, PK, and PP). Moderate length of branches was evident in four additional cases, subjects D, OJ, PO, and QP. The remaining 15 subjects revealed short branches indicating little intra-patient *env* diversity and apparent transmission of a single virus upon HIV infection. The topology and branching length of *env* quasispecies inferred by the NJ and ML methods suggested that 5 of 24 (20.8%) subjects were infected with multiple viral variants, and 15 (62.5%) subjects were infected with a single viral variant. The remaining 4 cases (16.7%) showed intermediate branching length suggesting that multiplicity of viral transmission in these cases is uncertain and requires additional analysis.

### Maximum and mean distances

The distribution of maximum and mean pairwise distances for *gag* and *env* sequences was studied to address whether applied evolutionary model(s) or specifics of viral distances can help to segregate HIV infections with transmission of single and multiple viral variants. Three types of maximum and mean pairwise distances, ML-corrected, K2P-corrected, and Hamming distances, as well as maximum and mean ML-corrected distances to MRCA sequence and K2P-corrected and Hamming distances to the consensus sequence, were analyzed.

**Figure 5:**
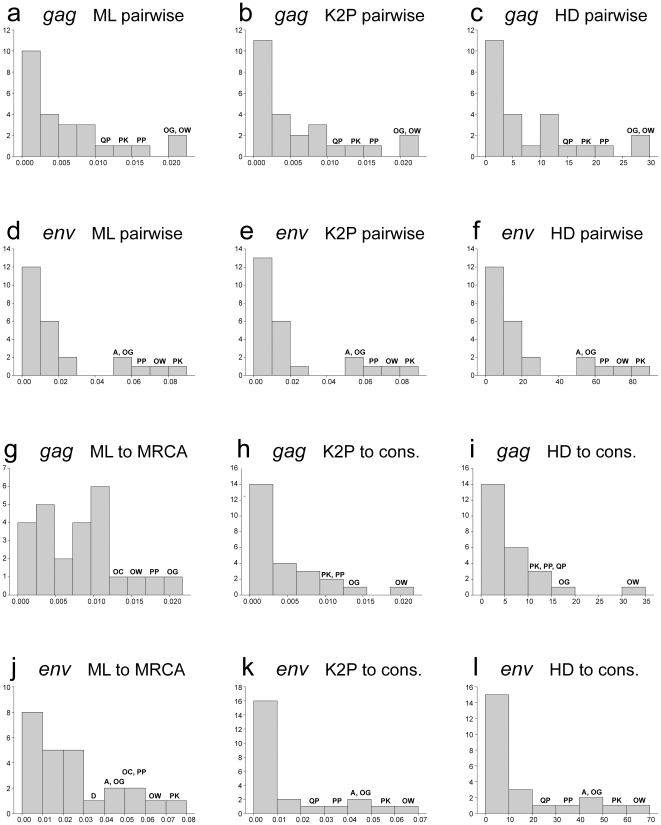
Distribution of HIV-1 subtype C *gag* and *env* maximum distances. y-axis denotes count of subjects per bin. x-axis denotes maximum distances. Single and double letters above the bins correspond to patients IDs. **A:** *gag* sequences, ML-corrected pairwise distances; **B:** *gag* sequences, K2P-corrected pairwise distances; **C:** *gag* sequences, Hamming distances; **D:** *env* sequences, ML-corrected pairwise distances; **E:** *env* sequences, K2P-corrected pairwise distances; **F:** *env* sequences, Hamming distances; **G:** *gag* sequences, ML-corrected distances to MRCA; **H:** *gag* sequences, K2P-corrected distances to consensus sequence; **I:** *gag* sequences, Hamming distances to consensus sequence; **J:** *env* sequences, ML-corrected distances to MRCA; **K:** *env* sequences, K2P-corrected distances to consensus sequence; **L:** *env* sequences, Hamming distances to consensus sequence.

The best segregation of HIV-1C infections was observed for maximum *env* pairwise distances including ML-corrected, K2P-corrected, and Hamming pairwise distances (Figs. 5D, 5E, and 5F). Consistent with the branching topology, the distribution of maximum *env* pairwise distances suggested transmission of multiple viral variants in five subjects, A, OG, OW, PP, and PK. The segregation of HIV-1C infections based on distribution of maximum *gag* pairwise distances was less discernible: only two subjects, OG and OW, showed separation from the remaining cases (Figs. 5A, 5B, and 5C). Two subjects, PK and PP, were on the right edge of the histogram tail but without separation from the main group of *gag* sequences. The continuous histograms with poor separation between cases, such as maximum ML-corrected distances to MRCA in Figs. 5G (*gag*) and 5J (*env*), produced the largest number of discrepant assignments. For example, ML-corrected distances to MRCA in *gag* generated assignments that were not congruent with other distance measurements in 7 of 25 cases of maximum distances and in 15 of 25 mean distances (see Cumulative Preliminary Summary discussion and Summary Table below). Similarly, little to no segregation was found for the maximum K2P-corrected (Figs. 5H – *gag* and 5K – *env*) and Hamming (Figs. 5I – *gag* and 5L – *env*) distances to the consensus sequence.

**Figure 6:**
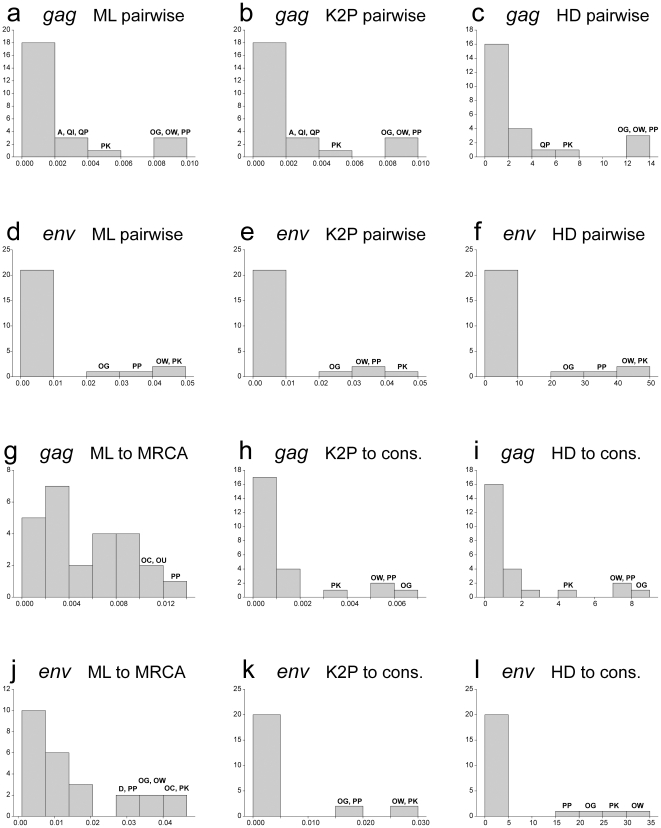
Distribution of HIV-1 subtype C *gag* and *env* mean distances. y-axis denotes count of subjects per bin. x-axis denotes mean distances. Single and double letters above the bins correspond to patient IDs. **A:** *gag* sequences, ML-corrected pairwise distances; **B:** *gag* sequences, K2P-corrected pairwise distances; **C:** *gag* sequences, Hamming distances; **D:** *env* sequences, ML-corrected pairwise distances; **E:** *env* sequences, K2P-corrected pairwise distances; **F:** *env* sequences, Hamming distances; **G:** *gag* sequences, ML-corrected distances to MRCA; **H:** *gag* sequences, K2P-corrected distances to consensus sequence; **I:** *gag* sequences, Hamming distances to consensus sequence; **J:** *env* sequences, ML-corrected distances to MRCA; **K:** *env* sequences, K2P-corrected distances to consensus sequence; **L:** *env* sequences, Hamming distances to consensus sequence.

The distribution of mean distances resembled the profiles of maximum distances. Interestingly, 7 out of 12 measurements (Figs. 6D, 6E, 6F, 6H, 6I, 6K, and 6L) segregated 4 samples, OG, OW, PP, and PK, out of five selected by the maximum *env* pairwise distances as cases with transmission of multiple viral variants. In addition, three of these 4 samples, OG, OW, and PK, were also separated from the main pool of samples by three mean *gag* pairwise distances (Figs. 6A, 6B, and 6C).

The decision thresholds (Table 2) were estimated based on the scale and distribution of maximum and mean distances, i.e., based on data presented in Figures 5 and 6. The decision thresholds differ between analyzed HIV-1 genes: *env*-related thresholds were 3 to 4 times higher than *gag*-based thresholds. The thresholds for maximum distances were 2 to 4 times higher than for mean distances. The thresholds for pairwise distances were higher than for distances to consensus sequence. The distribution specifics of ML-corrected distances to MRCA resulted in similar thresholds with ML-corrected pairwise distances. It is likely that this congruency between analyses of maximum and mean distances in segregation of viral sequences solidifies the evidence for the multiplicity of HIV transmission.

**Table 2:** Decision thresholds for transmission of single and multiple HIV-1 variants

	Maximum distances				Mean distances			
	*gag*		*env*		*Gag*		*env*	
Analysis	Multi	Single	Multi	Single	Multi	Single	Multi	Single
ML pairwise	>1.25%	<0.5%	>5%	<1%	>0.5%	<0.125%	>2%	<0.5%
K2P pairwise	>1.25%	<0.5%	>5%	<1%	>0.5%	<0.125%	>2%	<0.5%
Hamming pairwise	>15	<8	>50	<10	>5	<2.5	>20	<6
ML to MRCA	>1.2%	<1%	>4%	<2%	>0.5%	<0.125%	>2%	<0.5%
K2P to consensus	>1%	<0.5%	>3%	<1%	>0.25%	<0.063%	>1%	<0.25%
Hamming to consensus	>10	<5	>30	<10	>2.5	<1.25	>10	<3

Note: The following decision strategy was applied for each analysis of subject's maximum and mean distances within *gag* and *env*: A sample with value exceeding the “Multi” threshold was associated with transmission of *multiple* HIV-1 variants. A sample with value smaller than the “Single” threshold was associated with transmission of a *single* HIV-1 variant. A sample with value exceeding the “Single” threshold but less than the “Multi” threshold was considered *undetermined* in relation to multiplicity of HIV-1 transmission.

### Poisson fitness

The Hamming distance frequencies were analyzed by computing the best fitting Poisson distribution and evaluating results of the GOF. P-values of less than 0.05 indicate divergence from Poisson distribution and can be interpreted as transmission of multiple viral variants. The results of this analysis should be taken cautiously due to two limitations: the small number of sequences and the few relatively late (close to 50 days p/s) time points of sampling. Nevertheless it seemed important to compare results of the phylogenetic inference and viral diversity analyses with the new tool, Poisson-Fitter, which was developed for identification of transmissions caused by a single viral variant.

Four cases identified as transmission of multiple viral variants by both phylogenetic reconstruction and analysis of genetic distances (subjects OG, OW, PK, and PP) were also classified as multiple HIV infections in the Poisson-Fitter by GOF p-value of less than 0.05 for both *gag* and *env* sequences. Cases of HIV-1C infection considered “undetermined” by phylogeny and distance analyses (4 in *gag* analysis and 5 in *env* analysis) were also rejected as transmissions of single viral variant by the Poisson-Fitter method. However, the Poisson-Fitter rejected a few additional cases that were identified as transmissions of a single viral variant by phylogenetic and distance analyses: 3 in *gag* (subjects E, F, and QI) and 3 in *env* (subjects C, OU, and QI).

A comparison of phylogenetic inference, analysis of viral diversity, and the Poisson-Fitter analysis revealed overall good congruence between these methods for identification of HIV-1 transmission multiplicity. All cases that were identified as transmission of multiple viral variants or “undetermined” (implying that transmission of multiple viral variants cannot be excluded) were correctly rejected by the Poisson-Fitter through the GOF test. However, the Poisson-Fitter seemed to over-reject transmission of a single viral variant in some cases. This was observed for both *gag* (n = 10) and *env* (n = 16) sequences in subject QI, who was sampled during Fiebig stage IV at day 20 p/s. A few other cases that were identified as transmission of single viral variants by the phylogenetic and viral diversity analyses were rejected by the Poisson-Fitter either in *gag* (subjects E and F) or in *env* (subjects C and OU) analyses. This observation suggests that consistency of the Poisson-Fitter results for *gag* and *env* analysis produce reliable results of identification of multiplicity of HIV transmission, while discrepant *gag* and *env* results indicate a high level of uncertainty and warrant further analysis by alternative methods.

### Cumulative preliminary summary

**Figure 7:**
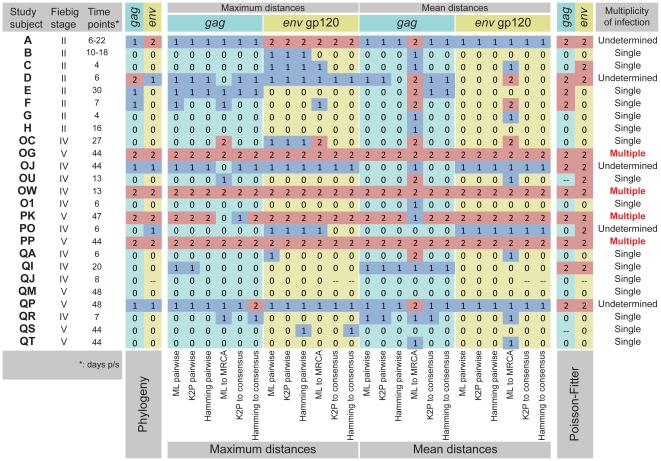
Summary Table of initial assessment for multiplicity of HIV-1C transmission. The Table includes the following sections: Study subjects with corresponding Fiebig stage and time of sampling in days p/s; Results of phylogenetic analysis, Maximum distances, Mean distances, Results of the Poisson-Fitter analysis, and Conclusion regarding multiplicity of HIV-1 transmission. Color coding of background: columns with *gag* results have light blue background, and columns with *env* results have light yellow background. Numeric coding: 0 – transmission of single viral variant; 1 – undetermined; 2 – transmission of multiple viral variants. Numeric coding of “1” and “2” are further enhanced by blue and light red colors. Each subsection of maximum and mean distance for both *gag* and *env* includes 6 columns with ML-corrected pairwise distances, K2P-corrected pairwise distances, Hamming distances, ML-corrected distances to MRCA, K2P-corrected distances to consensus sequence, and Hamming distances to consensus sequence. The last column “Multiplicity of infection” represents summary of initial analysis per subject indicating transmission of “Single” or “Multiple” viral variants in successfully resolved cases. The uncertain and non-congruent results are interpreted as “Undetermined” cases, and are subjects for detailed analysis by additional methods.

**Figure 8:**
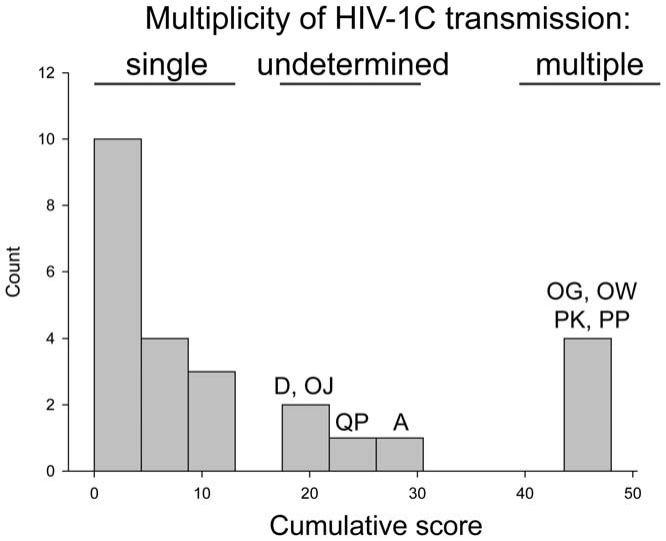
Distribution of cumulative score of initial assessment for multiplicity of HIV-1C transmission. y-axis denotes count of subjects per bin. x-axis denotes cumulative score. Single and double letters above the bins correspond to patient IDs. The cumulative score represents a sum per patient from data presented in the Summary Table in Figure 7. Three categories of HIV-1 transmission multiplicity include transmission of “single,” “undetermined,” and “multiple” viral variants.

The cumulative summary of results obtained by phylogeny, distance analysis, and the Poisson-Fitter is presented in Figure 7. The results are coded by “0” for transmission of single viral variant, “1” for transmission of undetermined number of viral variants (with additional blue coloring), and “2” for transmission of multiple viral variants (with additional light red coloring). The distribution of cumulative scores produced clear segregation of HIV-1C infections into groups that were associated with the multiplicity of viral transmission as “single,” “undetermined” and “multiple” (Fig. 8). The shape of cumulative score histograms provided better separation of single and multiple HIV-1C infection than any single method of analysis, highlighting the importance of multiple analyses in determining multiplicity of HIV-1 transmission.

The combined analysis of phylogeny, maximum and mean distances, and Poisson fitness suggested that transmission of multiple HIV-1C variants occurred in 4 (20% of resolved, or 16% of analyzed) cases, while transmission of a single viral variant occurred in 16 (80% of resolved, or 64% of analyzed) cases. Therefore, a conclusive decision on the multiplicity of HIV-1 transmission based on sampling within 50 days p/s was obtained for 80% of the analyzed cases. The multiplicity of HIV-1 transmission in the remaining five cases (20%), subjects A, D, OJ, PO and QP, was inconclusive and warranted further analyses.

### Detailed analysis of initially “undetermined” cases

Initial analysis based on the phylogenetic inference, analysis of viral diversity, and Poisson fitness analysis produced “undetermined” results for five subjects, A, D, OJ, PO, and QP. To resolve the multiplicity of HIV-1 infection in these cases, the following methods were applied: distribution of individual *gag* and *env* distances, shape analysis of highlighter plots, recombination analysis, and estimation of time to MRCA.

#### Distribution of individual HIV-1C *gag* and *env* distances

**Figure 9:**
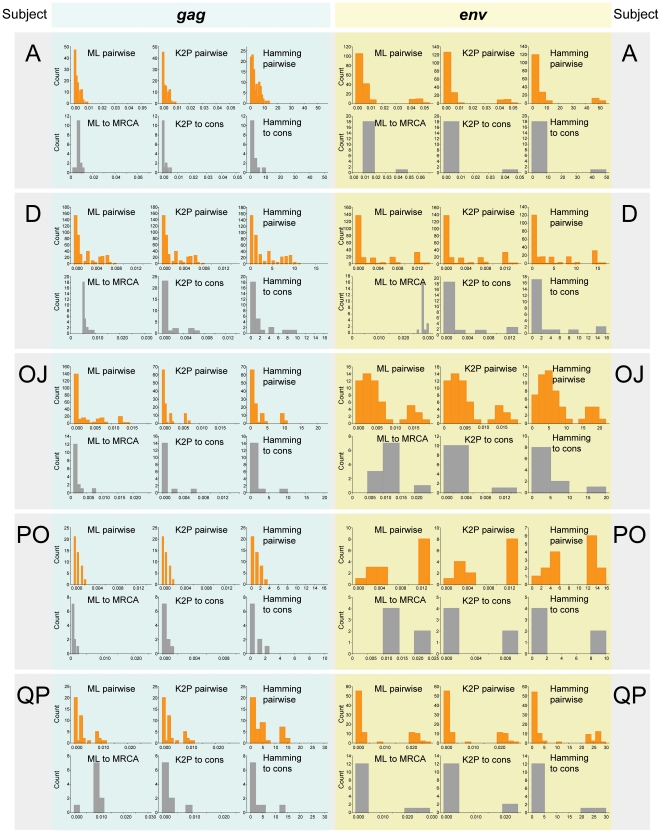
Distribution of individual HIV-1 subtype C *gag* and *env* distances for 5 “undetermined” cases. Patient IDs are shown on the left and on the right. All *gag* distances are shown on the left with light blue background. All *env* distances are shown on the right with light yellow background. Each gene/patient block includes 6 histograms with ML-corrected pairwise distances, K2P-corrected pairwise distances, Hamming distances, ML-corrected distances to MRCA, K2P-corrected distances to consensus sequence, and Hamming distances to consensus sequence. y-axis denotes count. x-axis denotes distances. Pairwise distances are shown in orange, and distances to MRCA and consensus sequences are shown in gray.

For individual sets of *gag* and *env* sequences in subjects A, D, OJ, PO, and QP, the distribution of pairwise ML-corrected, K2P-corrected, Hamming distances, ML-corrected distances to MRCA, and K2P-corrected and Hamming distances to the consensus sequence were analyzed (Fig. 9). In contrast to the previously described analysis that utilized only maximum and mean distances, all individual pairwise distances were included in the distribution analysis. Two subjects, A and PO, demonstrated distinct patterns for *gag* and *env* distance distribution, suggesting transmission of a single variant based on the *gag* distance distribution, but two viral variants were evident from the distribution of *env* distances. In contrast, three other subjects, D, OJ, and QP, showed congruence between *gag* and *env*, and the observed distribution patterns were consistent with transmission of multiple viral variants. Interestingly, *gag* ML-corrected distances to MRCA matched with other analyzed models in all 5 subjects. However *env* ML distances to MRCA matched the distribution of other analyzed distances only in 3 subjects, A, OJ, and QP, although they were skewed in subjects D and PO.

#### Highlighter plots

**Figure 10:**
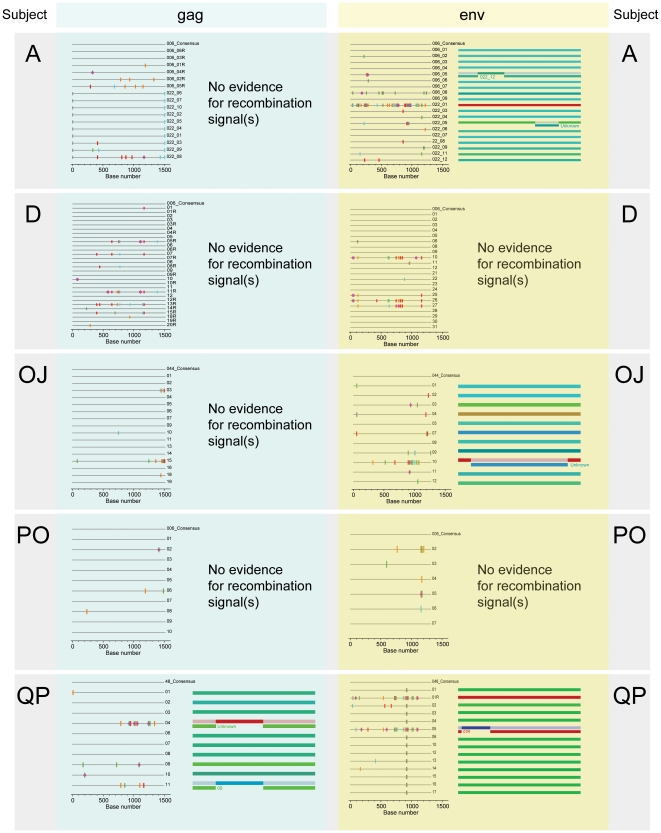
Highlighter plots and results of recombination analysis for 5 “undetermined” cases. Patient IDs are shown on the left and on the right. All *gag* data are shown on the left with light blue background. All *env* data are shown on the right with light yellow background. Highlighter plots were generated by the sequence visualization tool Highlighter. The recombination analysis was performed by RDP3. The order of analyzed sequences in the Highlighter plot corresponds to the order of sequences presented in the output from RDP3. The recombination events were identified in *gag* sequences 04 and 11 in subject QP, in *env* sequences 006_05 and 022_05 in subject A, sequence 10 in subject OJ, and in sequence 05 in subject QP.

The shape of highlighter plots (Fig. 10) was coherent with the branching topology of phylogenetic trees for *gag* (Figs. 1 and 2) and *env* (Figs. 3 and 4). The shape of the *gag* highlighter plot for subject PO was consistent with transmission of a single viral variant, while for subject QP it argued for transmission of multiple variants. The shape of the *gag* highlighter plots for subjects A, D, and OJ did not provide clear answers, leaving open the possibility of either transmission of a single variant followed by early diversification due to immune escape, or transmission of multiple viral variants. Thus, the multiplicity of HIV-1C infection for subjects A, D, and OJ was inconclusive based on the *gag* highlighter plots alone. The shape of the *env* highlighter plots suggested transmission of multiple viral variants in 4 of 5 subjects, and was rather inconclusive for subject PO. Taking into account only conclusive shapes, the combined results of the *gag* and *env* highlighter plots suggest transmission of a single viral variant in subject PO, and transmission of multiple viral variants in subjects A, D, OJ, and QP.

#### Recombination analysis

Analysis of *gag* sequences showed no evidence of a recombination signal in 4 of 5 subjects (Fig. 10). The potential recombination events were identified in two *gag* sequences from subject QP, sequences 04 and 11 (Fig. 10 and Table 3), supported by LARD and 3Seq analyses. Analysis of *env* sequences revealed the presence of recombinant sequences in 3 of 5 subjects (Fig. 10 and Table 3). Two *env* sequences with recombination signal in subject A were supported by MaxChi, SiScan, LARD, and 3Seq analyses (sequence 006_05), and SiScan and LARD (sequence 022_05). A single *env* sequence with recombination in subject OJ was supported by SiScan, LARD, and 3Seq analyses, while in subject QP the recombination signal was supported by BootScan, MaxChi, SiScan, LARD, and 3Seq analyses. We assumed that presence of recombinant sequences in the pool of viral quasispecies provides evidence for transmission of multiple viral variants, even if the source of recombination (parent sequence) has not been identified. Therefore, the recombination analysis supported transmission of multiple viral variants in 3 of 5 subjects: A, OJ, and QP.

**Table 3:** Recombination analysis, p-values (absence of p-value indicates no recombination event identified by the specified method)

		Methods of recombination analysis, p-values								
Subject (sequence)	HIV-1 gene	RDP	GENECONV	BootScan	MaxChi	Chimaera	SiScan	PhylPro	LARD	3Seq
**A**(006_05)	*env*	-	-	-	0.013	-	3.3×10^−3^	-	7.1x10^−7^	0.03
**A**(022_05)	*env*	-	-	-	-	-	4.2×10^−4^	-	1.4x10^−6^	-
**OJ** (10)	*env*	-	-	-	-	-	1.4×10^−3^	-	4.1x10^−8^	0.041
**QP** (04)	*gag*	-	-	-	-	-	-	-	6.8x10^−4^	0.042
**QP** (11)	*gag*	-	-	-	-	-	-	-	6.8x10^−4^	0.042
**QP** (05)	*env*	-	-	0.038	3.5x10^-3^	-	1.7×10^−8^	-	0.02	6.8x10^−3^

Time to MRCA (tMRCA) was estimated for *gag* and *env* sequences using BEAST v.1.5.4 [35], [40], and is shown in Table 4. The geometric means of tMRCA identified for both *gag* and *env* sequences were substantially outside the time of seroconversion estimated by Fiebig staging for all 5 subjects. The 95% lower and upper HPD parameters were in an extremely large range, which is not normally observed for transmission of a single viral variant. Although lower 95% HPD was within the time of seroconversion estimated by Fiebig staging for *gag* sequences in subjects OJ and PO, and for *env* sequences in subject QP, the obtained large ranges of HPD provide little justification for utilizing them as informative parameters for the analyzed set of sequences. Based on geometric means of the estimated tMRCA alone, transmission of multiple viral variants occurred in subjects A, D, OJ, PO, and QP.

**Table 4:** Estimated tMRCA, days.

		Estimated tMRCA			
		*gag*		*env*	
Subject	Estimated time p/s^1^ by Fiebig stage	geo mean (95% lower and upper HPD^2^)^1^	ESS^3^	geo mean (95% lower and upper HPD^2^)^1^	ESS^3^
A	6	193 (27; 753)	1,862	132 (20; 856)	533
D	6	194 (55; 528)	587	99 (21; 334)	1,095
OJ	44	174 (23; 1,039)	676	211 (50; 702)	1,248
PO	6	41 (2; 178)	1,253	208 (77; 458)	1,016
QP	48	351 (63; 1,520)	369	261 (44; 1,566)	519

1 Days post-seroconversion.

2 HPD is the highest posterior density interval, which represents the most compact interval on the selected parameter that contains 95% of the posterior probability. It is a Bayesian analog to a confidence interval.

3 ESS: Effective Sample Size – should be higher than 100, and characterizes the posterior distribution.

#### Summary of detailed analysis

**Figure 11:**
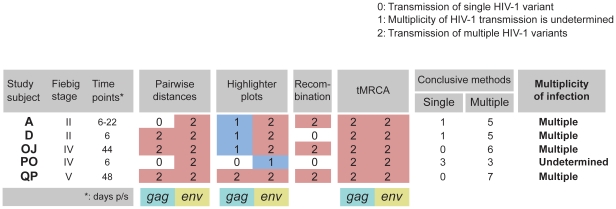
Summary table of second-step analysis for transmission of multiple HIV-1 variants in five subjects. The table includes the following sections: study subjects with corresponding Fiebig stage and time of sampling in days p/s; cumulative results of distribution of pairwise distances based on six analyses per gene (ML-corrected distances, K2P-corrected distances, Hamming distances, ML-corrected distances to MRCA, K2P-corrected distances to consensus sequence, and Hamming distances to consensus sequence); analysis of highlighter plots; cumulative results of recombination analysis; tMRCA; number of methods suggesting transmission of single and multiple viral variants; and conclusion regarding multiplicity of HIV-1 transmission. Numeric coding: 0 – transmission of single viral variant; 1 – undetermined; 2 – transmission of multiple viral variants. Numeric coding of “1” and “2” are further enhanced by blue and light red colors.

Due to some inconsistent results regarding multiplicity of HIV-1C transmission in 5 cases, we estimated how likely it is that transmission of multiple viral variants occurred based on the number of methods with conclusive results for each subject (Fig. 11). The results obtained in detailed analyses were weighted similarly to the summary table presented in Figure 7. As shown in Figure 11, all methods with conclusive results suggested transmission of multiple viral variantsin subjects OJ and QP. In subjects A and D, five methods suggested transmission of multiple viruses, while one method (pairwise distances and recombination analysis for subjects A and D, respectively) argued for transmission of single virus. In subject PO, the cumulative results were split with three methods suggesting transmission of single virus, and three methods suggesting transmission of multiple viral variants. Therefore, the cumulative results of detailed analyses suggested that four subjects—A, D, OJ, and QP—were infected with multiple viral variants, while multiplicity of HIV-1 transmission in one subject, PO, remained inconclusive.

### HIV-1C RNA load

We tested whether HIV-1 RNA load differs between subjects infected with single and multiple viral variants. Although subjects with transmission of multiple viral variants seemed to have higher HIV-1 RNA load at the time of sampling (median 5.75 log_10_ copies/ml; IQR 3.58–6.31) than subjects with single transmitted virus (median 4.89 log_10_ copies/ml; IQR 2.60–5.58), the difference did not reach statistical significance (p = 0.11; Mann-Whitney Rank Sum test).

### Summary of results

Transmission of a single viral variant was identified in 16 of 25 (64%; 95% CI 45% to 83%) analyzed cases. Transmission of multiple viral variants was confirmed in 8 of 25 (32%; 95% CI 14% to 50%) cases. For one subject, PO, we were unable to determine the multiplicity of transmission. Based on self-reports collected at enrollment, all HIV-1 transmissions in this study occurred heterosexually. Therefore, based on analysis of samples collected within 50 days p/s we estimate that the frequency of heterosexual transmission of a single viral variant in HIV-1 subtype C infection ranges from 64% to 68%, and that transmission of multiple viral variants ranges from 32% to 36% of HIV-1C infections.

## Discussion

The study demonstrated the utility of viral quasispecies analysis obtained within 50 days p/s for identifying multiplicity of HIV-1 subtype C infection. The suggested two-step approach was able to resolve 24 of 25 (96%) cases. The first step of the analysis was fast and straightforward, and was based on a combination of phylogenetic reconstruction, distribution of viral distances, and analysis of Poisson fit. Application of the first-step analysis resulted in successful assignment of multiplicity of HIV-1C infection in 20 of 25 (80%) cases as two extremes representing clear separation of single viral variant transmission from transmission of multiple viral variants. However “undetermined” results were produced in 5 (20%) cases due to inconsistency among the applied methods. The second step of the analysis attempted to resolve cases with “undetermined” initial results based on more detailed analysis that included intra-patient distribution of viral genetic distances, shape analysis of highlighter plots, recombination analysis, and estimation of tMRCA. Four of 5 initially “undetermined” cases were resolved in the second-step analysis.

The study highlights the complexity of early post-seroconversion evolution in HIV-1 subtype C infection among subjects. The study suggests that multiplicity of HIV transmission in the majority of HIV infections can be resolved by a combination of relatively simple analytical methods. However, a smaller fraction of about 20% of cases might require more sophisticated analyses. There was an evident discrepancy between some of the analyses in estimating multiplicity of HIV infection. The underlying possible reasons might include, but are not limited to, different sensitivity of evolutionary models used for distance correction, uncertainty in reconstruction of MRCA, and accelerated viral evolution within key epitopes in response to immune pressure from the host that can affect reconstruction of MRCA and subsequent estimation of tMRCA. These results indicate the imprecise nature of current methods and warrant further studies to better understand why different conclusions may be reached via different analyses. The observed complementary nature of applied multiple methods in identifying multiplicity of HIV transmission suggests the necessity of further development of alternative methodologies and bioinformatic techniques to improve the reliability of diagnostic and monitoring of the number of transmitted viral variants on a population level in the HIV/AIDS epidemic.

A combined use of viral quasispecies representing different genes (i.e., *env* and *gag*) was important for analysis of multiplicity of HIV transmission. Thus, a congruence between HIV-1C *env* gp120 V1-C5 and *gag* quasispecies supported the conclusion regarding multiplicity of HIV transmission. In contrast, discrepancy between viral genes might indicate the presence of early selection (immune) pressure within one but not another viral gene, and would argue for transmission of a single viral variant, as in the cases of subjects A and D. The gp120 V1-C5 sequences generally showed better segregation between transmission of single and multiple viral variants than HIV-1C *gag* quasispecies, apparently due to a higher viral diversity within *env* as compared with *gag*. For viral quasispecies obtained within 50 days p/s, the distribution of maximum pairwise ML-corrected distances was useful for separation of HIV-1C infections with transmission of single and multiple viral variants.

Knowledge of the multiplicity of HIV-1 transmission is a critical component of successful public health management of the HIV/AIDS epidemic, and advancement of HIV-1 transmission prevention. The multiplicity of HIV-1 transmission is strongly associated with the mode of viral transmission. Other factors, such as risk behavior, plasma HIV-1 RNA levels, and co-infections, can also affect both multiplicity of viral transmission and disease progression. Studies on estimating and monitoring of multiplicity of HIV-1 transmission are likely to reveal complex dynamics in the HIV/AIDS epidemic. Better understanding of underlying causes leading to transmission of multiple viral variants could improve public health strategies aimed at controlling and containing the spread of HIV. The extent of transmission of multiple viral variants in local epidemics should be taken into account in the design and testing of HIV vaccine candidates.

In summary, the study suggests a two-step strategy for identification of multiplicity of HIV infection based on sequences of viral quasispecies obtained within 50 days p/s. This approach enables the resolution of transmission of single or multiple viral variants in nearly all analyzed samples.
